# Toxicology and Disease/Cancer Therapy in Reactive Oxygen Species-Mediated Drugs and Treatments

**DOI:** 10.1155/2015/860563

**Published:** 2015-03-10

**Authors:** Hsueh-Wei Chang, Shao-Yu Chen, Li-Yeh Chuang, Sanjay Guleria

**Affiliations:** ^1^Department of Biomedical Science and Environmental Biology, Research Center of Excellence for Environmental Medicine, Cancer Center, Kaohsiung Medical University Hospital, Kaohsiung Medical University, Kaohsiung 80708, Taiwan; ^2^Department of Pharmacology and Toxicology, University of Louisville, Health Sciences Center, Louisville, KY 40292, USA; ^3^Department of Chemical Engineering and Institute of Biotechnology and Chemical Engineering, I-Shou University, Kaohsiung 84001, Taiwan; ^4^Division of Biochemistry and Plant Physiology, SK University of Agricultural Sciences and Technology, Chatha, Jammu 180009, India

Reactive oxygen species (ROS) induce or protect cellular oxidative damage. ROS are also known to play an important role in apoptosis, autophagy, endoplasmic reticulum stress, mitochondrial dysfunction, cell migration, and invasion. This special issue presents the recent advances in basic research and association studies of some natural products and chemical drugs for cancer and disease treatments.

The papers by M.-G. Lee et al. and H.-W. Huang et al. reported the antioxidant/tyrosinase suppression/wound repair properties and antiproliferative effect on oral cancer of* Cinnamomum osmophloeum* Kanehiraand* Cryptocarya concinna *Hance root extracts, respectively. Similarly, S.-C. Chien et al. reported the protective effect of Chinese herbal medicine “Jia-wei-xiao-yao-san” against chemical-induced hepatic fibrosis in rats.

Y. Fong et al. reported the antiproliferative and antiapoptotic effects on lung cancer cell lines of sirtinol, a sirtuin inhibitor. W.-C. Lee et al. reported that indoxyl sulfate (IS) induced oxidative stress and endothelial dysfunction in chronic kidney disease patients can be due to mitochondrial dysfunction and impaired biogenesis which can be reverted by treatment with antioxidants.

The papers by B. Huang et al. and P.-H. Chen et al. provided the findings on the upstream role of H_2_S and arsenic in modulating protein S-nitrosylation in endothelial cells and keratinocytes, respectively. W.-T. Chang et al. reported that epirubicin/progesterone combination is effective in increasing apoptosis and inversely decreasing autophagy on HA22T/VGH cells and can potentially be used to treat hepatocellular carcinoma.

The papers by S.-J. Wu et al. and P.-H. Chen et al. provided the cancer association studies of betel quid-consuming patients in terms of oxidative stress response genes such as polymorphisms for the human retinoic acid (RA) 4-hydroxylase (CYP26), the monoamine oxidase, and the cytochrome P450, family 26, subfamily B, polypeptide 1 (CYP26B1) genes.

Finally, the paper by Y.-R. Lin et al. introduced the proteomics technique for evaluating cytotoxicity of unmodified nano-Fe_3_O_4_ based on tandem mass spectrometry (LC-MS/MS) and T.-C. Cheng et al. provided the* in silico* virtual screening to discover specific inhibitors for intestinal* E. coli* *β*-glucuronidase.

*Hsueh-Wei*  *Chang* *Hsueh-Wei*  *Chang* *Shao-Yu*  *Chen* *Shao-Yu*  *Chen* *Li-Yeh*  *Chuang* *Li-Yeh*  *Chuang* *Sanjay*  *Guleria* *Sanjay*  *Guleria*

